# Metformin prolongs lifespan through remodeling the energy distribution strategy in silkworm, *Bombyx mori*

**DOI:** 10.18632/aging.101746

**Published:** 2019-01-13

**Authors:** Jiangbo Song, Guihua Jiang, Jianfei Zhang, Jieshu Guo, Zheng Li, Kaige Hao, Lian Liu, Zilin Cheng, Xiaoling Tong, Fangyin Dai

**Affiliations:** 1 State Key Laboratory of Silkworm Genome Biology, Southwest University, Chongqing 400716, China; 2 Key Laboratory for Sericulture Biology and Genetic Breeding, Ministry of Agriculture, College of Biotechnology, Southwest University, Chongqing 400716, China; * Equal contribution

**Keywords:** metformin, *Bombyx mori*, lifespan, energy distribution, AMPK-p53-FoxO pathway

## Abstract

Metformin is a hypoglycemic agent used clinically in the treatment of type 2 diabetics. In addition, metformin is being investigated as a potential geroprotector. Here, we investigated the effects of metformin silkworm lifespan and the underlying molecular pathways involved. We found that metformin prolonged the lifespan of the male silkworm without reducing body weight, which suggests metformin can increase lifespan through remodeling of the animal’s energy distribution strategy. Consistent with that idea, metformin reduced silk production and thus the energy devoted to that process. Metformin also increased fasting tolerance and levels of the antioxidant glutathione, and also activated an adenosine monophosphate-activated protein kinase-p53-forkhead box class O signaling pathway in silkworm. These results suggest that activity in this pathway may contribute to metformin-induced lifespan extension in silkworm by increasing stress resistance and antioxidative capacity while reducing energy output for silk product. The results also show that the silkworm is a potential useful animal model for evaluating the effects of small molecules with potential clinical utility.

## Introduction

Metformin was originally derived from a plant called goatsrue (*Galega officinalis L*), which is mainly found in southern Europe, southwest Asia and northwest China [1-4]. In ancient Egypt and medieval Europe, it was discovered that using goatsrue as a tea-like beverage relieved the diuresis and sweet halitosis that are now known to be typical symptoms of diabetes [1,5-7]. It was later found that the active ingredient galegine, also known as isoprene guanidine, could reduce blood glucose concentrations; however, the high toxicity of galegine precluded its clinical use [8]. Further analysis revealed that two biguanidine derivatives, metformin and phenformin, were less toxic [1,6,7], and ultimately. metformin was approved for the treatment of type 2 diabetes [1,9].

Metformin also lowered the incidence of several age-related diseases, including cancer, metabolic syndrome and cognitive disorders [10,11]. Because of its broad beneficial effects and minimal side effects, the ability of metformin protective effects against aging and aging-related diseases is being assessed in the Targeting Aging with Metformin (TAME) study approved by the U.S. Food and Drug Administration (FDA) in 2015 [12]. This is the first anti-aging drug study in humans to be initiated by National Institutes of Health (NIH) and FDA, and supported by the American Association for Aging Research (AAAR) [12]. At present, however, the mechanism underlying the proposed beneficial effects of metformin on lifespan extension are not well understood [13].

Metformin reportedly to extends the lifespan and delays the onset of aging-related diseases in the nematode *Caenorhabditis elegans* [14-17], mice [18], and rats [19], though not *Drosophila melanogaster* [20,21]. The current consensus is that metformin targets multiple cellular signaling pathways closely associated with aging-related ailments and lifespan, including inflammation, cellular senescence, stress defense, and autophagy [12]. One potential mechanism by which metformin may extend lifespan is that it mimics the effects of diet restriction by activating the principal energy sensor in cells adenosine monophosphate-activated protein kinase (AMPK) [16,18,22], though this remains to be tested.

The silkworm (*Bombyx mori*) is an increasingly popular experimental animal for use in life science research [23-26]. This is because the silkworm has a as clear genetic background, moderate body size, short life cycle, adaption to high density feeding in the laboratory, and relatively obvious boundaries between different developmental stages [27]. For these reasons, we used the silkworm to evaluate the effects of metformin on triggered molecular signals and pathways.

## RESULTS

### Metformin extends male silkworm lifespan

**Figure 1:**
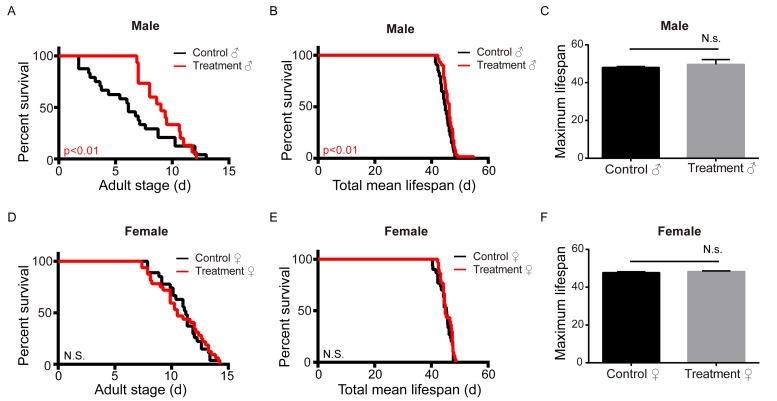
**Metformin increases the adult and total mean lifespan in the male silkworm.** (**A**) Adult stage (n=27), (**B**) mean total lifespan (n=30), and (**C**) maximum lifespan (n=3) of unmated female silkworms administered metformin (Treatment) or deionized water (Control). (**D**) Adult stage (n=24), (**E**) mean total lifespan (n=54) and (**F**) maximum lifespan of unmated male silkworms administered metformin (Treatment) of deionized water (Control). Bars depict the mean + SEM, *P < 0.05, **P < 0.01, ***P < 0.001.

To determine the effect of metformin on their lifespan, silkworms were fed fresh mulberry leaves with or without metformin. Our results show that male silkworms treated with metformin lived longer than the untreated control. Both the adult lifespan and the total mean lifespan of the male silkworm treated with metformin were significantly prolonged, though there was no increase in maximal lifespan. In addition, metformin had no significant effect on adult, total mean, or maximum lifespan in female silkworms (Figure 1A-F). The total mean and adult lifespans of the male silkworm were elongated by 1.2 days (2.68%) and 0.99 days (9.45%), respectively, as compared to control (Figure 1D, E). The metformin dose used was relatively low. It may be that a higher dose would elongate the lifespan of female silkworms. Alternatively, it may be that a sex-specific regulator is involved in the process of lifespan regulation in these animals.

### Energy distribution strategy is remodeled in metformin-induced lifespan extension

**Figure 2:**
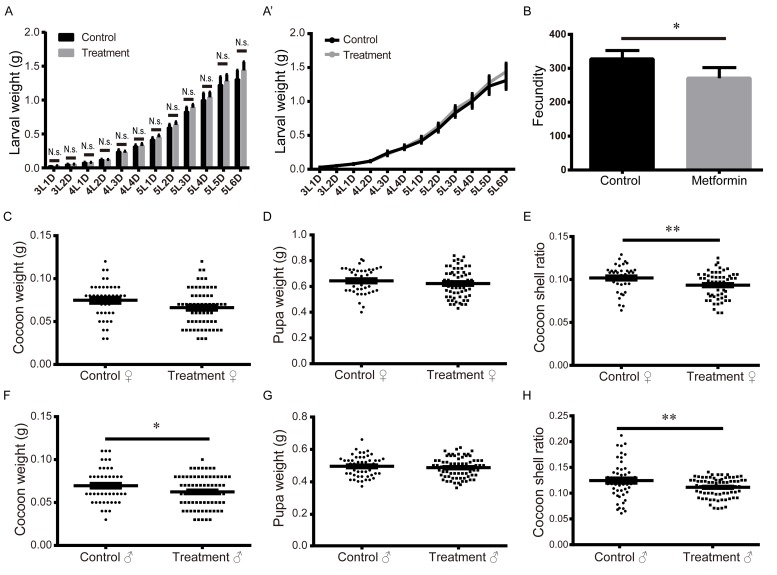
**Effects of metformin on larval weight, fecundity, pupal weight, cocoon weight, and cocoon-shell ratio.** (**A-A’**) Larval weights measured daily from L3D1 to L5D6. Bars and symbols depict the mean + SEM, n=5. (**B**) Fecundity. Bars depict the mean + SEM, n=6. (**C**) Female pupal weight, (**D**) cocoon weight, and (**E**) cocoon-shell ratio. Horizontal bars depict the mean ± SEM, n=61. (**F**) Male pupal weight, (**G**) cocoon weight, and (**H**) cocoon-shell ratio. Horizontal bars depict the mean ± SEM, n=75.

The energy distribution strategy is an important determinant of the lifespan of an organism. Evidences suggest that suppressing energy output is often sufficient to extend the lifespan [28-32]. To determine whether the energy distribution strategy was remodeled in metformin-induced lifespan extension, we compared body weight, silk output, and fecundity between silkworms in the treatment and control groups. The results showed that body weights did not significantly differ between the control and treatment groups from day 1 of the 3^rd^ instar to day 6 of the 5^th^ instar (Figure 2A, A’). The cocoon-shell ratio is the ratio between silk production and pupa weight, which is a key index of the scale of the energy distribution between silk production and the silkworm individual. Our results show that both the fecundity of the adult silkworm and the cocoon-shell ratio were significantly reduced in the metformin treatment group (Figure 2B-H). We therefore suggest that metformin-mediated lifespan extension may be achieved by rebalancing silk production and reproductive consumption. If so, this would indicate that there is a coupled zero-sum relationship among reproductive consumption, silk production, and survival maintenance in the metformin-induced lifespan extension in silkworm. AMPK is the direct target of metformin, and AMPK is also the key energy regulator. We therefore speculate that AMPK is key to metformin-induced energy redistribution.

### Metformin protects silkworm from environmental stress

**Figure 3:**
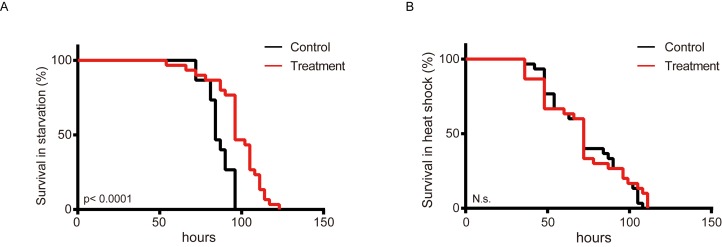
**Metformin increases starvation tolerance in the silkworm.** Survival curves showing percent survival over time among silkworms administered metformin (Treatment) or deionized water (Control) and exposed to (**A**) starvation or (**B**) a heated environment (37°C) (n=30).

Lifespan elongation is associated with amplified stress resistance in many organisms [33-36]. To determine whether metformin exerts protect effects against nutritional and thermal stress, metformin-treated and control silkworms were subjected to fasting and thermal stress (Figure 3A, B). The results show that metformin obviously increased the survival rate (by 13.46%) of silkworms subjected to feeding stress (Figure 3A), but did not increase thermotolerance (Figure 3B).

### Metformin enhances antioxidative properties

**Figure 4:**
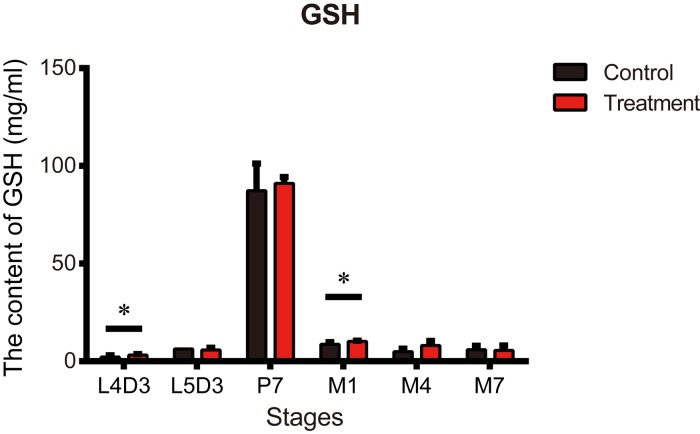
**Metformin increases the antioxidant content in silkworms at the P7 stage.** Glutathione (GSH) content measured at the indicated developmental stages in silkworms administered metformin (Treatment) or deionized water (Control). Bars depict the mean + SEM, n=9. *P < 0.05, **P < 0.01.

Metformin may also exert ergogenic effects that enhance the protective effects of an organism’s defense against attacks, leading to beneficial effects on lifespan [37]. Previous research suggests metformin may suppress cellular oxidative stress [15]. We therefore assessed the antioxidant activity of metformin in silkworms. GSH content is generally accepted as a crucial indicator antioxidative capacity. The GSH content of silkworms in the treatment group was significantly higher than in untreated controls on day 3 of the 4^th^ instar larva (L4D3) and day 1 of the adult stage (M1) (Figure 4). On the other hand, GSH content did not differ between the groups on day 3 of the 5^th^ instar larva (L5D3), day 7 of the pupal stage (P7), day 4 of the adult stage (M4), and day 7 of the adult stage (M7) (Figure 4).

### Lifespan extension is attributable via *AMPK-P53-FoxO* pathway

**Figure 5:**
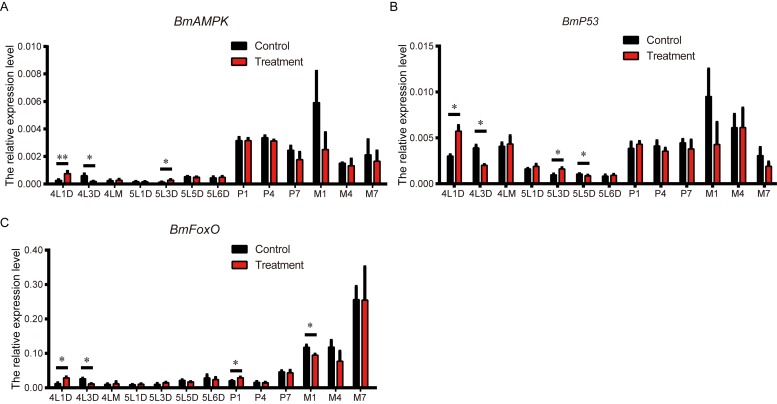
**Effects of metformin on the expression of *BmAMPK*, *Bmp53* and *BmFoxO*.** Expression levels of *BmAMPK*, *Bmp53* and *BmFoxO* at the indicated developmental stages in silkworms administered metformin (Treatment) or deionized water (Control) determined using real-time PCR. Bars depict the mean + SEM, n=9. *P < 0.05, **P < 0.01.

AMPK is a principal energy sensor in cells, but the geroprotective effects of low-dose metformin may be independent of the AMPK pathway [9]. *FoxO*, a key point of convergence of networks involved in regulating longevity, may be activated by *AMPK* [38]. Moreover, p53 and FoxO are reportedly required for metformin-induced growth inhibition and longevity, with p53 serving to bridge AMPK and FoxO [39]. Therefore, to clarify the signal transmission network involved in metformin-mediated lifespan extension, we compared the expression levels of *Bombyx mori* (*Bm*)*AMPK*, *Bmp53*, and *BmFoxO* metformin-treated and control silkworms. The level of *BmAMPK* expression in the treatment group was higher than control on L4D1, lower on L4D3, then higher again on L5D3 (Figure 5A). Expression of *Bmp53* in metformin-treated silkworms followed a similar time course from L4D1 to L5D3 then was lower than control on L5D5 (Figure 5B). As with *BmAMPK* and *Bmp53*, expression of *BmFoxO* the treatment group was higher than control on L4D1 and lower on L4D3 (Figure 5C). It was also significantly elevated on day 1 of the pupa stage (P1) but suppressed on M1, which is a substantial length of time after P1. The similar expression patterns of these three genes suggest that there is crosstalk and regulatory interaction among *AMPK*, *p53*, and *FoxO*, and that an *AMPK-p53-FoxO* pathway contribute to metformin-induced lifespan extension in the silkworm.

## DISCUSSION

Human life expectancy has increased over the past few years largely due to improvements in the treatment of aging-related diseases. Research into interventions for aging itself is certainly a new and revolutionary direction, at present. The TAME study, which has just been approved by the FDA, is the first major clinical study in humans, and it is anticipated that it will provide important information on the lifespan-extending effects of metformin [12]. The ability of metformin to lower blood glucose has been reported previously [1]. If it can be confirmed that, in addition to its beneficial effects in patients with type 2 diabetes, metformin is also involved in regulating aging and longevity, it could profoundly change the current treatment mode for aging-related diseases. It could result in a shift from treating each aging-related disease to targeting aging itself. Such a shift could contribute to the development of a new generation of drugs directly targeting the pathophysiological process of aging. Our study shows that metformin can prolong lifespan, thereby providing preliminary evidence supporting large clinical studies on lifespan elongation and highlighting the importance of examining the mechanism underlying the beneficial effects of metformin.

The anti-aging effects of metformin will likely involve secondary effects, such as an alteration in physiological energy distribution. In the case of the silkworm, reproductive output and silk production negatively correlate survival duration [32]. Our study demonstrated that metformin extends the silkworm lifespan by reshaping the energy distribution such that energy for silk output and reproduction is turned to survival maintenance. The past decade has seen fundamental advances in our understanding of the effects of dietary restriction, which has consistently been found to play a pivotal role in increasing longevity in both mammals and invertebrates [38]. However, the present results indicate that metformin-induced lifespan extension may not depend on dietary restriction, but instead depends on energy rebalance.

The transcription factor FoxO is an important regulatory target of AMPK and prolongs longevity by upregulating target genes involved in stress resistance [40]. Our results indicate that in the process of prolonging the silkworm lifespan, metformin increases both hunger tolerance and antioxidative capacity, and we speculate that FoxO may mediate these effects. To further characterize the potential mechanism of metformin-induced lifespan elongation, we compared the gene expression profiles of *BmAMPK*, *Bmp53*, and *BmFoxO* in silkworm. Given our observation that FoxO expression is increased on L4D1 and P1, we suggest that metformin may mediate antioxidant activity and fasting tolerance by triggering signal transduction via FoxO and its downstream targets. Moreover, the sequential changes in the expression levels of *BmAMPK*, *Bmp53* and *BmFoxO* suggest there are regulatory factors upstream of FoxO maintaining the appropriate expression level over time. Based on our results and earlier research [16,22], we speculate that metformin extends lifespan through activation of its direct target, AMPK, which promotes FoxO activity through modulation of p53. This AMPK-p53-FoxO signaling pathway is at least partially responsible for the effect of metformin on lifespan.

There are a wide variety of traditional medicinal materials in use in China, and there is an urgent to clarify their mechanisms so as to provide a physiological basis for their further development and utilization. Further investigation into the mechanism underlying the effects on aging of metformin, a derivative of goatsrue, is thus worth pursuing. To evaluate the beneficial effects of new potential drugs, it is necessary to gather specific knowledge from appropriate animal models [41]. The silkworm is a low-cost experimental animal useful for screening drugs. Thanks to its short lifespan and ease of genetic manipulation, the silkworm is becoming a useful model organism for testing the efficacy of molecules with potential to increase healthspan. Our results further confirm the utility of the silkworm for drug efficacy evaluation and preliminary drug screening.

In summary, our results suggest that the metformin exerts geroprotector effects in the silkworm by increasing antioxidative capacity and fasting tolerance, and by remodeling energy consumption distribution. However, it remains to be determined whether metformin-induced lifespan extension effect is conserved in other species.

## MATERIALS AND METHODS

### Metformin treatment

Commercial preparations of metformin were obtained from the Sigma-Aldrich (St. Louis, MO, USA). A 10-mM stock solution of metformin was prepared in sterile deionized H_2_O. After sterilization by filtration through 0.22 μm membranes, the stock solution was diluted to final concentrations of 0.1 mM, and stored at 4 °C. Each silkworm was fed mulberry leaves with 5μl 0.1 mM metformin (treatment group) or deionized water (control group).

### Silkworm strain and feeding conditions

Wildtype silkworm strain Dazao was obtained from the Silkworm Gene Bank at Southwest University and was maintained at 25°C with degression of relative humidity from approximately 75% to 50% over each 12 h light/12 h dark day. The silkworms were reared on fresh mulberry leaves throughout the entire larval stage [42].

### Lifespan assay

Throughout the entire life cycle, lifespan analysis was conducted at 25°C in an incubator with an optimal silkworm growth environment, as described previously [42]. Maximum lifespan refers to the upper 10% of the lifespan distribution. The survival condition of the silkworm specimens was checked of by monitoring the silk moth every 3 h, recording the time of death, and determining the mean and maximum lifespans.

### Measurement of body weight, and fecundity

The 4^th^ instar larvae from both the control and treatment groups were chosen randomly and divided into three groups for the measurement of daily body weight. The body weights of silkworms in each group were measured before feeding each day at the same time. Fecundity was determined by counting the number of progeny per female adult silkworm [42].

### Stress tolerance assay

To induce stress, silkworm specimens were heated at 37°C in the incubator. Survival was monitored during the stress period, and the time of death was recorded. Alternatively, silkworm specimens was fasted from day 1 of the 5^th^ instar larva. Survival was checked by confirming touch-provoked movement, and the time of death was recorded.

### Measurement of antioxidative properties

Glutathione levels were measured using test kits (GSH content assay kit) from Suzhou Comin Biotechnology Co. Ltd (Suzhou, China). The GSH content of a homogenate from an entire silkworm was measured according to the kit instructions.

### Reverse transcription-quantitative PCR (RT-qPCR)

Silkworms in the control and treatment groups were obtained from day 1 or 3 of the 4^th^ instar to day 7 of the moth stage. Total cellular RNA was isolated from three individuals using a rapid extraction Total RNA Kit (BioTeke Corporation, Beijing) according to the manufacturer’s instructions. First strand cDNA was synthesized from the total RNA samples using a PrimeScript™ RT Reagent Kit with gDNA Eraser (TaKaRa, Japan). RT-qPCR was performed using a CFX96 RealTime System (Bio-Rad, USA) with iTaq Universal SYBR Green Supermix (Bio-Rad, USA). Eukaryotic translation initiation factor 4A (BmMDB probe ID sw22934) is an optimally stable gene in silkworm [43]. Primer pairs targeted regions of the reference gene *sw22934*, *BmAMPK*, *Bmp53*, and *BmFoxO* specifically, and the relative expression levels of each gene were normalized to *sw22934* and calculated as 2^-△△CT^ [9]. The primers used for RT-qPCR are shown in Table 1. The qPCR conditions followed the manufacturer’s instructions.

**Table 1:** Primer sequences used for quantitative real-time PCR in this study.

Primer name	Sense sequence (5′→3′)	Antisense sequence (5′→3′)
*Bmsw22934*	TTCGTACTGCTCTTCTCG	CAAAGTTGATAGCAATTCCCT
*BmAMPK*	GCACTTGGGTATAAGGTCACAGAG	CGTTCGCCCGACAAAGACT
*BmP53*	GGGCAATACAACTTCAGCGTC	ACATCTGCGTCACGGCGA
*BmFoxO*	GCACAGGACAACAGGCTCACAC	GCTTGGCGTCGGGATTGA

### Statistical analysis

Statistical analyses were performed using GraphPad Prism 6 (GraphPad Software, La Jolla, CA, USA). Results were presented as mean ± SEM. The significance of the differences was analyzed using two-tailed Student’s *t* test, two-way ANOVA and log-rank test [44]. Values of P < 0.05 were considered statistically significant.
